# A Temporal Map of Coaching

**DOI:** 10.3389/fpsyg.2017.01352

**Published:** 2017-08-08

**Authors:** Tim Theeboom, Annelies E. M. Van Vianen, Bianca Beersma

**Affiliations:** ^1^Department of Work and Organizational Psychology, University of Amsterdam Amsterdam, Netherlands; ^2^Organization Sciences, Vrije Universiteit Amsterdam Amsterdam, Netherlands

**Keywords:** coaching, Trans Theoretical Model of Change, awareness, willingness to change, ability to change, planning, integration of learnings

## Abstract

Economic pressures on companies, technological developments, and less stable career paths pose potential threats to the well-being of employees (e.g., stress, burn-out) and require constant adaptation. In the light of these challenges, it is not surprising that employees often seek the support of a coach. The role of a coach is to foster change by facilitating a coachees’ movement through a self-regulatory cycle with the ultimate aim of stimulating sustained well-being and functioning. While meta-analytic research indicates that coaching interventions can be effectively applied to assist employees in dealing with change, the current literature on coaching lacks solid theoretical frameworks that are needed to build a cumulative knowledge-base and to inspire evidence-based practice. In this conceptual analysis, we examine the coaching process through a temporal lens. By doing so, we provide an integrated theoretical framework: a temporal map of coaching. In this framework, we link seminal concepts in psychology to the coaching process, and describe which competencies of coachees are crucial in the different stages of change that coaching aims to bring about. During the preparatory contemplation stage, targeting coachees’ awareness by enhancing their mindfulness and environmental receptiveness is important. During the contemplation stage, coachees’ willingness and perceived ability to change are central competencies. We propose that coaches should therefore foster intrinsic goal orientation and self-efficacy during this stage. During the planning stage, coaches should focus on goal-setting and implementation intentions. Finally, during the maintenance/termination stage, stimulating coachees’ reflection is especially important in order to help them to integrate their learning experiences. The framework delineated in this paper contributes to the understanding of coaching as a tool to assist employees in dealing with the challenges of an increasingly dynamic work-environment and yields concrete suggestions for future theory development and research on coaching.

## Introduction

The current work environment is characterized by rapid technological developments, a shift toward knowledge-based work and less stable career paths (Savickas et al., 2009). Simultaneously, individuals in western societies attach more value to self-actualization and optimal well-being and functioning in both their work and personal lives (Joshanloo, 2013). Taken together, changes in work and self-actualization needs require constant adaptation and professional development on the part of employees, which poses multiple challenges to them. First, people in general tend to favor the status-quo and familiarity over change and uncertainty (Boswell et al., 2014) which means that they tend to avoid rather than anticipate change. Second, people often lack the self-regulatory knowledge, skills and attitudes needed for effective change (Baumeister and Heatherton, 1996). Third, personal and professional goals (e.g., spending more time with family versus getting a promotion) may cause complex dilemmas and work-family conflicts (Frone et al., 1992). In the light of these challenges and the associated health risks, it is not surprising that employees often seek the support of a coach.

Coaching has been defined as a ‘result-oriented, systematic process in which the coach facilitates the enhancement of life experience and goal-attainment in the personal and/or professional life of normal, non-clinical clients’ (Grant, 2003, p. 254). The role of a coach is to foster change by ‘facilitating a coachees’ movement through a self-regulatory cycle’ (Grant, 2003, p. 255) with the ultimate aim of stimulating sustained well-being and functioning (Grant, 2003, 2013). In other words, coaching is basically about fostering self-directed changes in coachees in order to make them feel and function better.

While meta-analytic research (Theeboom et al., 2014; Jones et al., 2016) indicates that coaching interventions can be effectively applied as a change methodology, current studies on coaching (e.g., Evers et al., 2002) do not reveal the processes through which these outcomes are attained. This is unfortunate, because knowledge of these processes is crucial for the evidence driven development of coaching interventions (Theeboom et al., 2014).

Of course, research in clinical, social, and organizational psychology offers many insights into competencies that are related to change. Currently however, insights into how exactly coaches can strengthen these competencies are fragmented, because no clear theoretical framework exists that links this rich knowledge base to the coaching process. Therefore, when and why coaches should use which strategies is largely a black box. Interestingly, in their Theory of Team coaching, Hackman and Wageman (2005) proposed that it could be useful to view coaching through a temporal lens because different coaching interventions may be relevant and effective during different stages of the change process. Specifically, they stated that ‘coaching interventions are more effective when they address issues a team is ready for at the time they are made’ and that ‘ill-timed interventions may actually do more harm than good’ (p. 275). Likewise, the idea that viewing the coaching process through a temporal lens could provide useful insights into coaching of individuals is reflected in the work of both Passmore (2011) and Grant (2012), who suggested that the Trans Theoretical Model of Change (TTM, Prochaska and Velicer, 1997) could be a useful framework for coaching.

Originally developed to conceptualize and study behavior change (see Prochaska, 2013; for a comprehensive review of the model) and to assess individuals’ readiness to change across multiple health behaviors such as smoking, physical exercise, and stress reduction (Hall and Rossi, 2008), the TTM posits that behavior change occurs in five distinct stages of change (see below). As such, the temporal lens offered by the TTM enables building a clear framework related to which types of coaching strategies are helpful at which stages of the coaching process, and why this is the case. This is the goal of the current paper. In our conceptual analysis, we offer research evidence where available and address possible avenues for future research where necessary. By integrating the “stages of change” perspective as outlined in the TTM with important concepts in different disciplines of psychology, the current paper can facilitate theory development, research on coaching, and coaching practices.

## The Trans Theoretical Model of Change and Its Value for Understanding Coaching

The TTM posits that behavior change occurs in five distinct stages of change: precontemplation, contemplation, preparation, action, and maintenance. Furthermore, this model is built on the notion that behavior changes as individuals contemplate on and weigh the gains and losses of their behaviors. The precontemplation stage is aimed at becoming aware of a need for change and increasing the pros of behavior change (e.g., stop smoking). The contemplation stage is meant to further explore one’s motivation and to decrease the cons of behavior change (e.g., giving up favorite habits associated with smoking). The preparation stage involves setting specific goals and making plans for action, which may involve concerns about possible failure. The action stage is about setting the first small steps toward the attainment of goals and to start exercising and practicing desired behaviors. In this stage individuals have to work hard to keep from returning to earlier stages. The maintenance stage concerns preventing relapse and consolidating progress (Prochaska and Velicer, 1997; Grant, 2012).

Although the TTM was created in the context of the cessation of unhealthy behaviors and is focused on individuals’ readiness for behavioral change, we argue that the basic change principles underlying this model clearly suit the reality of coaching, which is also aimed at changing behaviors (Grant, 2003, 2012). That is, individuals who come to coaching may be in varying stages of change and change involves a gradual movement through specific changes (Prochaska and DiClemente, 2005). Some coaching authors have done some groundwork in linking the TTM to coaching. For example, Passmore (2011) has offered some specific guidelines for interventions in the different stages. According to Passmore, coaches could help coachees to explore their wider values, beliefs, and the impact of the behavior of others in the first stage, while coaches could help coachees to plan for coping if falling back in the maintenance stage. Likewise, Grant (2012) argued that – in a work context – coachees can become aware of their own needs and those of the organization through a 360° feedback intervention in the first stage, which will help them to select their goals. Furthermore, he posited that the stages outlined by the TTM are particularly useful to assess a coachees’ change readiness.

Although we view these preliminary suggestions as important steps toward developing a temporal map of coaching, we believe that a solid integration of the stages as outlined in the TTM and important concepts in clinical, social, and organizational psychology could enhance theory development and research on coaching, and facilitate coaching practices. The definitions of coaching presented above refer to concepts such as goals and self-regulation, which are central for coach-supported change to occur. These concepts are also at the heart of seminal theories in psychology. For example, a general and influential theory on explaining human behavior, and thus relevant for behavior change, is the Theory of Planned Behavior (Ajzen, 1991). This theory argues that individuals’ behavior is goal-directed and guided by behavioral intentions (the effort individuals are planning to exert in order to perform the behavior) if this behavior is under volitional control (individuals’ beliefs in their self-efficacy to execute a certain behavior). Other important theories, such as Self-Determination Theory (SDT) (Deci and Ryan, 2000), Goal-setting Theory (Locke and Latham, 2002), and Self-efficacy Theory (Bandura, 1977), all underline the importance of goals, behavioral intentions, and self-efficacy for individual behavior (change) as delineated in the Theory of Planned Behavior. In what follows, we address these theories and their main concepts when discussing the successive stages of the coaching process.

In this paper, we build on the TTM by developing a temporal map of the coaching process. In addition, based on seminal theories in clinical, social, and organizational psychology we identify essential competencies that coachees should develop or strengthen in each stage of the coaching process in order to move from one stage to another. We particularly argue that attainment of core competencies in one stage is a prerequisite for competency development and change progress in the next stages. By identifying relevant focal competencies for each stage, specific interventions can be developed or refined and research could test their effectiveness and underlying assumptions. This can ultimately contribute to the development of evidence-based practices in coaching that foster employee welfare and strength, and decrease occupational risk factors.

In the next section, we discuss the stages of the temporal map of coaching^1^ in more detail. For each stage of the coaching process we suggest two focal competencies that are evidently relevant in that stage. However, note that the competencies that we propose are by no means intended to be exhaustive, but rather emerge from current theories and research in psychology. Furthermore, we address previous research (in the broader psychological literature and/or in the specific coaching literature) that relates these competencies to the proximal (individual self-regulated change) and distal goals of coaching (individual well-being and functioning). Finally, we provide suggestions for future research on coaching and coaching practice.

## Preparatory Contemplation: Developing Awareness

**Table 1** presents an overview of the main goals in each stage of the coaching process and the proposed focal competencies that should be addressed in these stages. We label the first stage of the coaching process as preparatory contemplation rather than precontemplation. The TTM describes the first (precontemplation) stage as a stage in which no intention to change (“why would I stop smoking?”) develops into an intention to change (Prochaska and Velicer, 1997). However, when – in the context of work and careers – individuals are contacting a coach they are to a certain extent (being) motivated to start the coaching process, whether this is due to experienced difficulties, an executive coaching program offered by the organization, or questions regarding their career.

**Table 1:** The main goals and focal competencies during each stage of the TTM.

Stage	Main goal	Focal competencies
Preparatory contemplation	Development of awareness	• Mindfulness
		• Environmental receptiveness
Contemplation	Exploring the willingness and perceived ability to change	• Intrinsic goal orientation
		• Self-efficacy
Planning	Planning for change	• Goal-setting
		• Implementation intentions
Maintenance/termination	Integration of learnings	• Reflection


The main goals of the preparatory contemplation stage in coaching is to identify individual and context factors that are the impetus for the coaching and to come to the realization that there is a need to change. The primary task of a coach during this stage is to raise coachees’ awareness of the individual and organizational drives for change. This awareness is important as it is a prerequisite for successful follow-up steps: the further exploration of drives, the setting of goals, and developing action plans in line with these drives. Moreover, we believe that this awareness will smoothen the change process (see also Boyatzis, 2006). Note that this stage would be especially (but not exclusively) important for so-called ‘unvoluntary’ coachees (e.g., who are sent by their organization), because raising awareness of the factors that led to coaching could enhance a coachee’s intrinsic motivation to change (see below) and his or her engagement in the coaching process. Hence, the interventions of the coach are aimed at developing or strengthening coachees’ awareness competencies. Two competencies in particular could enhance awareness of individual- and organizational drivers of change, respectively: mindfulness and environmental receptiveness.

## Mindfulness

The construct of mindfulness has been adopted from the Buddhist tradition (Hayes and Feldman, 2004) and was originally introduced in the psychological literature by Kabat-Zinn (1990). Mindfulness consists of two basic components (Bishop et al., 2004, p. 1) the self-regulation of attention that allows for increased recognition of present mental experiences, and an open, curious, and accepting orientation toward those experiences.

Developing coachees’ mindfulness could help coachees to become aware of their needs and desires in three ways. First, mindfulness enables individuals to openly observe and reflect on their own thoughts, emotions, and behaviors. Second, the inward focus enhanced by mindfulness may help coachees to explore their own values and motivations (Hayes et al., 2006) which increases the chance that they set self-concordant goals (i.e., goals that are consistent with individuals’ interests and core values; Sheldon and Elliot, 1999) in the subsequent stages. Third, mindfulness could enable coachees to make a distinction between issues that they can potentially solve and issues that they can’t influence, and to stay focused on the solvable issues (Hayes et al., 1999).

Alhough a lot has been written about mindfulness and coaching (e.g., Passmore and Marianetti, 2007), we know of only two independent pilot studies that directly investigated the potential synergy of mindfulness meditation and coaching interventions. Both studies found that mindfulness can be succesfully combined with coaching interventions and has the potential to amplify the effectiveness of those interventions (Collard and Walsh, 2008; Spence et al., 2008). Additionaly, previous research on mindfulness outside the coaching literature has shown that mindfulness is positively related to several aspects of change (the proximal goal of coaching) and to functioning and well-being (the ultimate goals of coaching). For example, mindfulness meditations decrease anxiety and negative affect by reducing cognitive distortions (thinking errors), self-set demands, and anticipated consequences of negative events (Sears and Kraus, 2009). Likewise, mindfulness fosters benign stress attributions (Weinstein et al., 2009), such as when the stress arises from being in a power position (Boyatzis et al., 2006), and it advances attentional control (Jha et al., 2007), mental and physical health (Chiesa and Serretti, 2009; Hofmann et al., 2010), and academic and workplace performance (Beauchemin et al., 2008). In addition, mindfulness may not only be helpful by fostering self-awareness in the preparatory contemplation stage, but may have several additional benefits in the following stages of the coaching process. For example, mindfulness will also be beneficial during the maintenance stage, because the attentional control associated with mindfulness is an important precondition for reflection (Cox et al., 2010), which in turn is an important antecedent of learning (Dewey, 1933). Hence, mindfullness may not only help the coachee to recognize what he/she truly needs and desires, and to set goals accordingly, but may also help to sustain learning from previous experiences.

## Environmental Receptiveness

Drawing on literature on epistemic motivation (an individual’s desire to develop a thorough understanding of a situation) in social and organizational psychology (e.g., Van Kleef et al., 2009) and on receptiveness in cross-cultural and communication research (e.g., Chen et al., 2010; Leung and Chiu, 2010), we conceptualize environmental receptiveness as the ability and willingness to receive environmental information, such as information about the thoughts and behaviors of significant others, what is needed and expected to function on a job (referent information), the extent to which role requirements are met (appraisal information), and information about the quality of the relationships with others (relational information: Miller and Jablin, 1991; Bauer et al., 2007).

A lack of environmental receptiveness often lies at the heart of a need for coaching. Generally, phenomena such as selective attention and change blindness make people less able to detect significant changes timely (Simons and Rensink, 2005). Moreover, much of the organizational information about what the organization expects or desires from its employees is hidden or ambiguous rather than open or clear. To give an example, misinformation about the (“soft” or informal) requirements for getting a promotion in an organization (e.g., getting along with other managers) often leads to frustration for both employees (‘but I did tick all the boxes for a promotion’) and their direct supervisors who have a hard time maintaining a perception of procedural justice. This lack of awareness of organizational (and social) needs, opportunities and constraints can leave coachees in the blind. Hence, interventions aimed at enhancing a coachees’ environmental receptiveness could help coachees to deal with, and maybe even prevent problems associated with the highly dynamic and complex nature of current organizational environments.

There is currently no research investigating the role of environmental receptiveness in coaching. However, research outside the field of coaching has shown that two elements underlying environmental receptiveness are positively related to self-regulated change, well-being and functioning, namely openness to experience (“an interest in varied experience for its own sake” McCrae, 1987, p. 1259) and epistemic motivation, the willingness to actively seek information in order to develop a rich and accurate understanding of the world (Amit and Sagiv, 2013).

Specifically, LePine et al. (2000) found that openness to experience predicted decision quality during a problem solving task in which the rules for correct decisions changed frequently. In addition, research on epistemic motivation evidenced that individuals who are motivated to seek alternative coping strategies for dealing with stressful situations feel more in control of these situations, apply a wider variety of strategies, and adapt more successfully (Cheng, 2003).

Research has also shown that information-seeking is one of the main tactics through which organizational newcomers can proactively deal with the uncertainty they experience during organizational entry (Ashford and Black, 1996; Kim et al., 2005; Ren et al., 2014). Newcomers who seek more information about what is required to function on the job and on how they are functioning adapt more easily due to more role clarity and social acceptance (Bauer et al., 2007).

All in all, we propose that in the preparatory contemplation stage, coaching interventions should be focused on raising coachees’ awareness of individual and organizational drivers of change by enhancing coachees’ competencies of mindfulness and environmental receptiveness. Furthermore, we propose that these coaching interventions in the preparatory contemplation stage will increase the effectiveness of coaching interventions in subsequent stages of the coaching process.

## Contemplation: Exploring the Willingness and Perceived Ability to Change

The contemplation stage revolves around reevaluating the self and the environment and the pro’s and cons of change (decisional balancing) in order to be able to make a firm commitment to action before taking the first steps toward change (Prochaska, 2013). During this stage, a coach helps coachees to clarify their own needs and values such that they can develop goals (Burke and Linley, 2007) and action plans (in the following stage) that are self-concordant, that is, congruent with their inner values and needs. In other words, coaches could use the contemplation stage to help coachees to develop an intrinsic (rather than extrinsic) goal orientation. Furthermore, in this stage it is important that coachees perceive themselves as able to change. As such, a coach could also use this stage to help coachees to explore and develop their self-efficacy (the belief in one’s ability to succeed in specific situations; Bandura, 1977).

## Intrinsic Goal Orientation

Self-Determination Theory (Deci and Ryan, 1985) states that all humans strive for the fullfilment of three basic needs: autonomy, competence, and relatedness. Autonomy refers to a sense of volition and self-endorsement of behavior, competence refers to support for the efficacy of autonomously selected goals, and relatedness concerns the sense of being cared for and connected to others. These needs play a crucial role for the types and strength of people’s motivation (Ryan et al., 2011).

According to SDT, intrinsic goals are aligned with the satisfaction of basic needs, whereas extrinsic goals are driven by external sociocultural norms associated with coercion, fear, or shame. People who are oriented toward intrinsic goals focus more on the content of an activity itself (e.g., an excercise) than on the contingencies following it (e.g., a reward), which foster their commitment and persistence, and ultimately their performance and well-being (Vansteenkiste et al., 2007).

According to Deci and Ryan (2008), “SDT provides empirically informed guidelines and principles for motivating people to explore experiences and events, and from that reflective basis, to make adaptive changes in goals, behaviors, and relationships. Because the issues of intrinsic motivation and of creating a climate conducive to volitional and lasting change are central to all psychotherapies, the principles of SDT are not only useful in informing therapeutic content but also have relevance across varied interventions and techniques” (p. 186). Although coaching differs from therapy in several ways (e.g., Hart et al., 2001; Grant, 2014), goal orientations can be expected to be equally important to coaching because all coaching engagements involve volitional and lasting adaptation and are essentially goal driven (Spence and Oaedes, 2011; Grant, 2012).

Only one study on coaching addressed coachees’ orientation toward intrinsic goals and found that the coaching intervention GROW (Goals, Reality, Options, Way forward; Whitmore, 2010) increased the development of self-concordant goals. However, an abundance of research outside of the context of coaching has shown that an intrinsic (rather than extrinsic) goal orientation is positively related to change, well-being, and functioning. For example, research in educational and sports settings indicates that an intrinsic goal-orientation fosters effective self-regulation. Students who are intrinsicly motivated show lower drop out of high school (Vallerand et al., 1997), are more deeply engaged in learning activities, show more persistence, and perform better than students who are less intrinsically motivated (Vansteenkiste et al., 2006). Also, athletes who have an intrinsic goal-orientation are more likely to engage in task-oriented coping (dealing with the problem at hand) whereas athletes with an extrinsic goal-orientation tend to disengage from their stressors (Smith et al., 2010). Furthermore, striving for self-concordant goals positively affects adjustment, identity development, and academic performance (Sheldon and Houser-Marko, 2001). Finally, setting self-concordant goals was found to promote (subjective) well-being irrespective of cultural context (Sheldon et al., 2004).

## Self-Efficacy

Self-efficacy has been defined as the belief in one’s ability to succeed in specific situations (Bandura, 1977). Although Bandura originally described self-efficacy as a domain-specific construct (e.g., self-efficacy for athletic performance), others introduced the concept of generalized self-efficacy that refers to an overall estimate of a person’s ability to mobilize the cognitive and motivational resources needed to deal with challenges in life (Judge et al., 1998).

Self-efficacy is a key component in the coaching process because it is a necessary basis for approaching problems from a new perspective, developing creative solutions, and experimenting with new skills and behaviors (Popper and Lipshitz, 1992). Hence, in order for coaching to be effective, it should be aimed at stimulating self-enhancing causal attributions (i.e., ascribing success to one’s own effort or skills rather than external circumstances) and building feelings of competence (Moen and Skaalvik, 2009; Moen and Federici, 2012). Self-efficacy will support coachees’ ownership of and responsibility for the adaptation process.

Several studies have investigated how coaching can influence self-efficacy. For example, a study by Evers et al. (2002) found that managers who received coaching scored higher on generalized self-efficacy than a control group. Likewise, Moen and Skaalvik (2009) found that a coaching intervention increased generalized self-efficacy for both a group of CEOs and middle managers as compared to their respective control groups. As one of the most frequently studied variables in the fields of social and organizational psychology, self-efficacy by itself has been extensively linked to coping behaviors (e.g., Devonport and Lane, 2006) and academic (Multon et al., 1991) and work-related performance (Stajkovic and Luthans, 1998). Furthermore, meta-analyses that studied self-efficacy as a component of higher order constructs such as Core Self-Evaluations (CSEs, consisting of self-efficacy, self-esteem, locus of control, and emotional stability; Judge and Bono, 2001) and Psychological Capital or PsyCap (consisting of self-efficacy, optimism, hope and resilience; Luthans and Youssef, 2004) have also found positive relationships with adaptation, well-being, and performance (e.g., Kammeyer-Mueller et al., 2009). Therefore, we propose that coaching interventions aimed at enhancing coachees’ intrinsic goal-orientation and self-efficacy are essential in the contemplation stage as they will increase the effectiveness of coaching interventions in subsequent stages of the coaching process.

## Planning for Change

In the planning stage (Passmore, 2011), commitments are made and there is an intention to quickly move into action. This stage is similar to what the TTM labels as the preparation stage, which refers to a readiness to change, the setting of goals, and the making of commitments with regard to new (health) behaviors (Prochaska, 2013). In the context of coaching, coaches can help coachees to review and select chosen options, and to clarify and refine goals. A first task for coaches during this stage is to facilitate coachees to further specify their intrinsic goals. However, ‘good intentions have a bad reputation’ (Gollwitzer, 1999, p. 439), meaning that goal intentions alone do not necessarily lead to effective actions. Instead, in addition to goals, people need to formulate implementation intentions that specify how they will deal with potential obstacles in order to ensure that goals can be attained. We discuss two focal competencies that could be addressed during the planning stage: setting goals and forming implementation intentions.

## Goal-Setting

According to Goal-setting Theory (Locke and Latham, 2002), goal-setting refers to the conscious guidance of moment to moment behavior toward a (consciously or unconsciously) desired end-state (Gollwitzer, 1999). Whereas SDT (Deci and Ryan, 1985) specifically focuses on the content of goals and the degree to which this content aligns with basic human needs and an individual’s interests and values, Goal-setting Theory mostly focuses on how goals are set and framed after goal-content has been determined.

Goal-setting Theory (Locke and Latham, 2002) and the relevance of helping coachees to set workable and effective goals in particular have been widely recognized in the coaching literature. Most definitions of coaching include the word ‘goal-focused’ (e.g., Grant, 2003) emphasizing that coaching is in essence a goal-focused activity (Gregory et al., 2011; Grant, 2012). Setting goals has proven to be essential in making the changes needed to narrow the gap between a current situation and a desired end-state (Heckhausen et al., 2010).

Goal-setting Theory has undoubtedly been one of the most influential theories in coaching research and practice. Most of the empirical work on coaching has been conducted in the context of coaching methodologies that are heavily goal-driven such as the GROW model (see above) of coaching (Whitmore, 2010) and solution-focused coaching interventions that direct a coachee’s attention to a desired future situation (Berg and Szabo, 2005; Theeboom et al., 2016). To give an example, Moen and Skaalvik (2009) showed that executives who underwent coaching achieved an increase in goal specificity, elicited more feedback regarding their progress, and had clearer strategies for achieving their goals than executives in a control group. Moreover, meta-analytic research shows that goal-driven approaches to coaching can be effective for improving self-regulation, coping, well-being, and performance (Theeboom et al., 2014).

Outside of the specific coaching research, the literature on goal-setting is arguably one of the richest literatures in the field of organizational psychology and studies have demonstrated that goal-setting is strongly related to change, well-being, and performance (e.g., LePine, 2005). Further, the general literature on goal-setting provides specific guidelines for practitioners as research has shown that goal-setting is most effective when goals are SMART (specific, measurable, achievable, realistic, time-targeted; Rubin, 2002) and when goals are challenging, proximal in time, focused on mastery (‘I want to become better than before’) rather than performance (“I want to outperform others”; Pintrich, 2000), and when goal-setting is accompanied with seeking feedback about the progress that is made in realizing the goals (Locke and Latham, 2002; LePine, 2005). For example, participants in health programs who set specific goals and self-monitored their progress were found to show better attendance and less dropout (Annesi, 2002), to eat healthier, and to lose more weight (Shilts et al., 2004) than participants who did not set specific goals.

## Implementation Intentions

The preparation stage revolves around planning, which can be described as “a prospective self-regulatory strategy, a mental simulation of linking concrete responses to future situations that can be used to replace spontaneous *in situ* reactions by pre-planned details of action implementation and detailed strategies for coping with anticipated obstacles” (Sniehotta et al., 2005, p. 566). Effective planners should not stop once a goal is set (Gollwitzer, 1999) but they should also proactively think about how their goal-intentions can be translated into behavior and how they can deal with the potential obstacles that could hinder goal-striving (Sniehotta et al., 2005). In other words, planning is about forming implementation intentions which have the form of ‘if situation X is encountered, then I will perform the goal-directed response Y’ (Gollwitzer and Sheeran, 2006). Coaching seems to be especially suited to forming implementation intentions: the client-centered dyadic nature of coaching allows for in-depth conversations about (how to deal with) the unique social and practical obstacles that coachees can encounter while translating their intentions into actual behavior.

As coaching is about fostering actual changes rather than merely strengthening intentions to change, especially in organizational contexts where individuals and organizational stakeholders expect some sort of return on their investments (i.e., a positive balance between gains and costs), it is surprising that there is a lack of research on the role that coaching can play in forming implementation intentions. However, several meta-analytic investigations and experimental studies outside the domain of coaching have shown that adding implementation intentions to goal intentions considerably contributes to goal-achievement (Koestner et al., 2002; Gollwitzer and Sheeran, 2006; Bayer et al., 2010). Individuals who combine strong goal intentions with implementation intentions show better progress toward short-term and long-term personal goals than individuals who merely have strong goal intentions (Koestner et al., 2002). In addition, forming implementation intentions benefits well-being because it increases people’s self-efficacy in dealing with setbacks and shields them from feelings of anxiety and ego-depletion (i.e., the loss of mental resources necessary for self-control and willpower) during goal-striving (Bayer et al., 2010).

Altogether, setting goals and forming implementation intentions are central in the planning stage of the coaching process and these competencies are necessary for the making and maintenance of actual changes. Hence, we propose that coaching interventions aimed at enhancing coachees’ competencies to set goals and to form implementation intentions in the planning stage will increase the effectiveness of coaching interventions in the next stage of the coaching process.

## Maintenance and Termination: Integration of Learnings

Individuals in the maintenance stage have already made important changes but still require to invest some effort in preventing relapse (Prochaska, 2013). This need for relapse prevention becomes less salient (although not obsolete) during the termination stage in which new behaviors have become automatic and have replaced the old patterns of behavior. In the termination stage, individuals disengage from effortful change. In the context of coaching, the most important role of a coach during this stage is to help the coachee to solidify their learnings and to integrate new behaviors into their day-to-day lifes in order to ensure the ‘transfer of coaching.’ A competency that has been inseperably linked to this kind of integrative (“transformative”; Mezirow, 1997) learning is reflection.

## Reflection

Reflection concerns ‘the pondering, reviewing, and questioning of experiences’ (Gallimore et al., 2014, p. 269). Several influential theories, such as Kolb’s (1984) Experiential Learning theory and Mezirow’s (1997), Transformational Learning view reflection as a mediator between an experience and learning. Hence, all genuine and profound learning is the result of reflecting on experiences (Dewey, 1933). Schön (1983) distinguishes two main forms of reflection: reflection-in-action (the immediate “thinking on one’s feet” in uncertain situations; Cox, 2013) and reflection-on-action which happens after the event. The latter seems most relevant to coaching since coaches are expected to be seldomly present when coachees are actually ‘in action.’

Theories on learning and reflection are highly important for coaching (Gray, 2006). First, learning is crucial for the long-term effectiveness of coaching. The return on investment of coaching is much higher when coachees have learned from the coaching experience that their self-regulatory capacities are enhanced and they are able to solve similar problems in the future. In terms of a chinese proverb: ‘Give a man a fish and he will eat for a day, teach him how to fish and he will eat for a lifetime.’ Actually, several roles that coaches typically play substantially overlap with teaching roles (Gray, 2006), namely providing information, serving as a resource, assessing learners’ needs and competencies, locating resources or securing new information, setting up learning experiences, working with learners as a sounding board for ideas, and helping learners to develop a positive attitude toward learning and self-directed inquiry (Jarvis, 1995).

While the importance of reflection (on action) has been addressed in the coaching literature (e.g., Burke and Linley, 2007; Cox, 2013), we are not aware of any studies that directly investigated the impact of reflection on (long-term) coaching effectiveness. There is only one study that showed that enhancing the antecedents of reflection, such as mindfulness, in combination with coaching is more effective for goal-attainment than coaching alone (Spence et al., 2008). Moreover, there is some empirical research on reflection in the domain of health education (see Mann et al., 2009). This research indicates that reflection, and collaborative reflection (such as reflection that occurs in the dialog between a coach an a coachee) in particular, is key to deep-level learning. Furthermore, it is argued that reflective practice can be facilitated through creating a “safe atmosphere, mentorship and supervision, peer-support, and time to reflect” (p. 614). Hence, we propose that coaching interventions aimed at enhancing coachees’ competencies to reflect on their experiences in the maintenance and termination stages will increase the transfer of coaching.

## Discussion

The main aim of this conceptual analysis was to outline a theoretically rich and empirically driven temporal map for coaching interventions. Such a map is useful for both coaching practioners and scholars alike. For coaching practioners, a temporal map can help to determine focal points that could facilitate the change process and subsequentially, to decide which interventions will be most fruitful to help the coachee to move through the different stages. For scholars, considering the coaching literature through the lens of a temporal map helps to develop theory on the coaching process and to identify directions for future research.

Based on the TTM we delineated a temporal map of coaching and based on seminal theories and research in clinical, social, and organizational psychology we identified crucial competencies that coachees should develop or strengthen throughout the different stages of the coaching process. We posited that in the preparatory contemplation stage, the main task of a coach is to foster the coachees’ awareness of both his/her own needs and drives for change by enhancing mindfulness and those of the environment by enhancing environmental receptiveness. In the contemplation stage, the main task of the coach is to help the coachee to explore both his/her willingness to adapt by enhancing the focus on intrinsic (self-concordant) goals and his/her perceived ability to adapt by enhancing self-efficacy. In the planning stage, the main task of the coach is to facilitate the coachee to prepare for action by assisting them to set goals according to the principles of Goal-setting Theory and to develop implementation intentions that can help coachees to foresee and proactively cope with potential obstacles. Finally, we posited that in the maintenance and termination stages, the main task of coaches is to help coachees to reflect on their experiences and learning in order to increase the chance that coaching has a lasting impact.

We emphasize that the suggested focal competencies are not intended to be exhaustive and that future theoretical and empirical work is needed to reveal other important competencies that should be addressed in the different stages and to further refine the temporal map we outlined in this paper. As our review of the relevant literature for each stage points out, this might be especially relevant for the preparatory contemplation and maintenance/termination stages, which, up to now, have largely been ignored in the coaching literature.

In addition to proposing focal competencies per stage, we emphasize that the development or strengthening of these competencies in one stage are a prerequisite for coaching effectiveness in follow-up stages. Specifically, we propose that exploring one’s willingness and ability to change and developing an intrinsic goal orientation and self-efficacy in the contemplation stage will be doomed to fail if coachees lack mindfullness and environmental receptiveness. Likewise, the setting of goals and implementation intentions in the planning stage will be hard to realize when coachees lack an intrinsic goal orientation and self-efficacy. Finally, reflection in the maintenance stage will be superficial and insufficient for transfer of change when coachees are unable to set clear goals and implementation intentions. Althogether, we propose that the competencies together are the foundation of an individual’s sustained self-regulatory capacity, which is the ultimate goal of coaching.

Our temporal perspective on the coaching process requests the further development of a temporal theory of coaching that may be rooted in, and extend, existing theories such as the Conservation of Resource Theory (COR; Hobfoll, 2002) and the Broaden-and-Build Theory of Positive Emotions (B&B; Fredrickson, 2001). COR theory states that individuals are motivated to protect and strengthen themselves through the acquisition, maintenance, and replenishment of internal resources (e.g., self-efficacy) and external resources (e.g., organizational support) needed to cope with and adapt to environmental conditions (Hobfoll, 2002). COR theory articulates several assumptions that are highly important in the context of coaching. First, COR theory argues that resources are malleable and can be increased or decreased by individuals themselves, and thus, are accessible to interventions. Second, COR theory proposes that resources do not exist in a vacuum, but rather develop and co-exist in an ecological system with strong interrelationships called resource caravans (Hobfoll, 2011). Identifying resource caravans is highly relevant for the development of series of coaching interventions. Finally, COR theory suggests the existence of ‘gain spirals,’ which occur when an individual is able to cope successfully with environmental conditions and strengthens his or her resources in turn. The reciprocity of resources and outcomes into gain spirals is particularly important for the timing and phasing of coaching interventions. A coach may create a gain spiral by using an intervention that promotes competencies of an intrinsic goal orientation and self-efficacy in the contemplation stage which leads to immediate success and then applying follow-up interventions that build on this success.

Broaden-and-Build Theory of Positive Emotions postulates that positive emotions broaden peoples’ scope of thoughts and actions or thought-action repertoires. Further, it proposes that these broadened thought-action repertoires stimulate experimentation, risk taking and innovative behaviors, which result in the discovery of novel strategies that can be used to adapt effectively to the environment and to build the resources needed to deal with challenges in the future (Fredrickson and Joiner, 2002). This “build”-component of B&B theory also includes a reciprocal mechanism similar to the gain spirals described in COR theory. Successful competency development in one stage of the coaching process induces positive emotions that will lead to broadened thought-action repertoires in a next stage.

Besides a need for more theory development along the lines suggested here, we envision several specific issues that could be addressed in future research on coaching. Below, we shortly discuss three of those issues: (1) the assessment of where a coachee is in the change process (2) a coachee’s movement through the different stages of change and (3) the coaching approaches and interventions that are particularly suitable in the different stages.

## Research Agenda

Coaching does not necessarily starts when a coachee is in the preparatory contemplation stage. It is possible that a coachee has already gone through the preparatory contemplation and contemplation stages by him or herself (or with the help of another coach, mentor or manager) and seeks help for translating earlier discoveries about (individual and environmental) drives for change into action (by developing suitable goals and forming implementation intentions in the planning stage). Likewise, it is also possible that a coachee seeks the help of a coach in the maintenance stage, in order to allow him- or herself to reflect on previous experiences. These examples show that in order for a coach to be effective, he or she needs to be aware of where the coachee is in the process of change (Grant, 2012). Therefore, a potentially interesting avenue for future research would be to develop and investigate diagnostic tools that can help to asesss where the coachee stands in the process. A promising starting point may be to adapt existing questionnaires, such as for example the Readiness To Change Questionnaire (Rollnick et al., 1992), for use in a coaching context.

Another (and related) question that remains open for future research is when and to what degree coaches should be targeted at ‘taking a step-back’ rather than moving forward through the stages. To give an example, consider a coachee who has been unsuccessful in attaining a goal (finishing a project) and fails to fix this problem by vigorously setting goals and forming implementation intentions. In this case, a potentially fruitful direction for a coach could be to assist the coachee in stepping back into the preparatory contemplation and/or contemplation stages in order to find out to whether the coachee is aware of all relevant environmental factors, whether his or her goals are self-concordant, and whether he or she is self-efficacious in undertaking effective action (before setting goals and forming implementation intentions). After all, other theories related to individual-level change such as the Intentional Change Theory (ICT) postulate that change is often a non-linear and discontinuous process (Boyatzis, 2006). We hope that future studies will explore a possible integration of the temporal map of coaching developed in this paper and ICT, as they could mutually strengthen each other. Specifically, the ‘discoveries’ that are, according to ICT, central to sustainable individual-level change can be linked to the coaching stages we discussed here. As an example, the ‘ideal self and personal vision’ discovery (see Boyatzis and Akrivou, 2006) fits well with the contemplation stage, as both entail a motivational component. As another example, the ‘learning agenda and plan’ discovery fits well with the planning stage. Integrating specific suggestions for interventions as provided by ICT with our temporal map of coaching could help coaches to develop these interventions. The ‘personal balance sheets’ (Boyatzis, 2006) are a good example of how ICT-based interventions can be incorporated in coaching practice.

This brings us to our final recommendation for future research. Future studies could investigate which specific coaching interventions are best suited to target the focal competencies outlined for each stage of the coaching process. Currently, most approaches to coaching described in contemporary textbooks (Cox et al., 2010; Palmer and Whybrow, 2014) originate in therapeutic interventions (e.g., cognitive-behavioral coaching, motivational interviewing, narrative coaching). However, coaching differs from therapy on many levels (e.g., the scope of the problems discussed, the target population, the duration and the degree of goal-orientedness of the process) and coaching interventions should differ accordingly (Grant, 2014). The focal competencies we have outlined may help to identify, develop, and investigate tailored coaching interventions.

To give an example, we discussed how enhancing a coachees’ self-efficacy could be important during the contemplation stage. According to Social Cognitive Theory (Bandura, 1977) self-efficacy beliefs are acquired via four informational sources: personal performance accomplishments, vicarious learning, social persuasion, and physiological and affective states. Future research could investigate how coaches can address these informational sources in order to enhance a coachees’ self-efficacy. For instance, a coach could emphasize personal performance accomplishments by asking a coachee to tell about previous success experiences related to the problem and/or to assist a coachee in seeking opportunities for successful accomplishments. Or, a coach with expertise in leadership can provide examples of successful leadership behaviors to inspire vicarious learning. Finally, a coach could engage in social persuasion by giving compliments and encouragements (e.g., ‘you have been a key employee in this team for years, I am sure you’ll do great as a manager). Similarly, future research could investigate which interventions are useful to enhance mindfulness (e.g., mindfulness meditation; see Spence et al., 2008 for an example) and environmental receptiveness (e.g., perspective taking exercises) in the preparatory contemplation stage, setting SMART goals and forming implementation intentions in the preparation stage, and reflecting on experience (e.g., using diaries and written reflections) in the maintenance/termination stage.

In order to address these issues (the stage of change assessment, the movement between stages, and the utility of specific interventions for each stage), we encourage a combination of quantitative and qualitative research methodologies. Coaching often is a highly individualized and complex process (Passmore and Theeboom, 2015) and coachees often work in complex organizational settings that may influence their change process. Therefore, quantitative research alone is unlikely to capture the complexity and the richness of experiences and working environments of coachees (Grant, 2013). It is only by combining different methodologies that we can attain a comprehensive understanding of coaching as a change methodology.

## Conclusion

We are the first to acknowledge that the temporal map outlined in this paper is truly a starting point and could benefit from input from scholars in different fields of psychology, coaching, and practitioners alike. We have argued that a temporal map of the coaching process is needed because it will promote theory and research on coaching which, in turn, will result in the development of timely and tailored coaching interventions. In this paper, we have outlined various focal competencies that coaches could address in the different stages of the coaching process. Moreover, we have proposed that the timely targeting of competencies will enhance the effectiveness of coaching interventions in subsequent stages. While the competencies are based on previous coaching literature and seminal theories and research in clinical, social, and organizational psychology, our propositions require empirical tests to extend and refine our temporal map. We hope that the temporal map of the coaching process delineated in this paper may be a starting point for scholars and practitioners who are dedicated to understanding and developing coaching as a change-methodology.

## Author Contributions

The three authors discussed the main purpose and design of this conceptual analysis together. TT wrote a first draft of the main part of this paper. AV added text to the first draft and BB also added text in a first round. The authors discussed the text together and each of the authors critically revised the first text in a second round in a similar order as in the first round. The paper went through several subsequent rounds (and discussions) in the same order as in the first and second round. Finally, all authors approved the current version and agreed that all authors are accountable for all aspects of the work, including issues of accuracy and integrity of the writing process and written text.

## Conflict of Interest Statement

The authors declare that the research was conducted in the absence of any commercial or financial relationships that could be construed as a potential conflict of interest.

## Footnotes

1 We excluded the action stage from our framework because this is the stage in which the coachee acts without the coach being present.
